# Trends and Inequalities in the Incidence of Acute Myocardial Infarction among Beijing Townships, 2007–2018

**DOI:** 10.3390/ijerph182312276

**Published:** 2021-11-23

**Authors:** Jie Chang, Qiuju Deng, Moning Guo, Majid Ezzati, Jill Baumgartner, Honor Bixby, Queenie Chan, Dong Zhao, Feng Lu, Piaopiao Hu, Yuwei Su, Jiayi Sun, Ying Long, Jing Liu

**Affiliations:** 1 Department of Epidemiology, Beijing An Zhen Hospital, Capital Medical University, Beijing 100029, China; 13601247286@163.com (J.C.); qiujudeng@126.com (Q.D.); deezhao@vip.sina.com (D.Z.); hupiaopiao1996@163.com (P.H.); heyiyao@sina.com (J.S.); 2 Beijing Institute of Heart, Lung and Blood Vessel Diseases, Beijing 100029, China; 3 National Clinical Research Center of Cardiovascular Diseases, Beijing 100029, China; 4 Beijing Municipal Key Laboratory of Clinical Epidemiology, Beijing 100069, China; 5 Beijing Municipal Health Commission Information Center, Beijing 100034, China; guomoning@wjw.beijing.gov.cn (M.G.); lufeng@wjw.beijing.gov.cn (F.L.); 6 Department of Epidemiology and Biostatistics, School of Public Health, Imperial College London, London W2 1PG, UK; majid.ezzati@imperial.ac.uk (M.E.); q.chan@imperial.ac.uk (Q.C.); 7 MRC Centre for Environment and Health, School of Public Health, Imperial College London, London W2 1PG, UK; 8 Abdul Latif Jameel Institute for Disease and Emergency Analytics, Imperial College London, London W2 1PG, UK; 9 Regional Institute for Population Studies, University of Ghana, Accra P.O. Box LG 96, Ghana; 10 Institute for Health and Social Policy & Department of Epidemiology, Biostatistics and Occupational Health, McGill University, Montreal, QC H3A 1G1, Canada; jill.baumgartner@mcgill.ca (J.B.); honor.bixby@mcgill.ca (H.B.); 11 School of Urban Design, Wuhan University, Wuhan 430072, China; suyuwei@whu.edu.cn; 12 School of Architecture, Tsinghua University, Beijing 100084, China; 13 Key Laboratory of Eco Planning & Green Building, Ministry of Education, Beijing 100084, China; 14 School of Architecture and Hang Lung Center for Real Estate, Tsinghua University, Beijing 100084, China

**Keywords:** acute myocardial infarction, incidence, temporal trends, inequality

## Abstract

Acute myocardial infarction (AMI) poses a serious disease burden in China, but studies on small-area characteristics of AMI incidence are lacking. We therefore examined temporal trends and geographic variations in AMI incidence at the township level in Beijing. In this cross-sectional analysis, 259,830 AMI events during 2007–2018 from the Beijing Cardiovascular Disease Surveillance System were included. We estimated AMI incidence for 307 consistent townships during consecutive 3-year periods with a Bayesian spatial model. From 2007 to 2018, the median AMI incidence in townships increased from 216.3 to 231.6 per 100,000, with a greater relative increase in young and middle-aged males (35–49 years: 54.2%; 50–64 years: 33.2%). The most pronounced increases in the relative inequalities was observed among young residents (2.1 to 2.8 for males and 2.8 to 3.4 for females). Townships with high rates and larger relative increases were primarily located in Beijing’s northeastern and southwestern peri-urban areas. However, large increases among young and middle-aged males were observed throughout peri-urban areas. AMI incidence and their changes over time varied substantially at the township level in Beijing, especially among young adults. Targeted mitigation strategies are required for high-risk populations and areas to reduce health disparities across Beijing.

## 1. Introduction

Ischemic heart disease is a leading cause of death worldwide and in China [1,2]. Acute myocardial infarction (AMI) is a serious manifestation of ischemic heart disease and is often fatal. The incidence of AMI in Western countries has decreased primarily due to the implementation of effective prevention strategies [3,4]. However, limited data on temporal trends for AMI incidence are available for low- and middle-income countries. Beijing is one of the most populous global cities. In recent decades, this city has experienced rapid economic and population growth and subsequent social and environmental changes, such as air pollution, rapid urbanization and lifestyle change, that have been correlated to cardiovascular risk in other populations [5,6]. However, the recent temporal trends in AMI incidence in Beijing, and their magnitude, remain unclear.

Research on spatiotemporal variations of cardiovascular disease (CVD) rate at the small-area level have important implications for developing an effective public health and policy response and has attracted attention [7,8,9]. However, small-area studies on geographic variations and long-term changes in the incidence of AMI are limited [10], especially within a large, rapidly changing global city. With its highly heterogeneous physical and social environments, Beijing provides an ideal setting to investigate the geographic variations in the incidence of AMI at a finer spatial resolution (e.g., at the township level).

Using annual data on AMI events extracted from the Beijing Cardiovascular Disease Surveillance System (BCDSS) covering a decade from 2007 to 2018, we aimed to explore temporal trends and empirically quantify geographic variations in AMI incidence at the level of Beijing’ s townships.

## 2. Materials and Methods

### 2.1. Study Setting

Beijing, which is China’ s capital, covers a total area of 16,410 km^2^ and comprises six districts located in the city’s urban core and ten districts, considered peri-urban areas. Districts can be further divided into townships. We conducted a geographic analysis at the township level, which is the smallest administrative unit in China. Given the changing administrative boundaries of some townships over time, 307 townships with consistent boundaries during the study period were included in the analysis (Figure S1). The median permanent population of a township was 33,903, while the 10th and the 90th percentiles population were 10,614 and 86,895, respectively.

### 2.2. Data Sources

AMI cases were identified through the BCDSS, which links routinely collected records in the Beijing Hospital Discharge Information System (HDIS) and the Beijing Vital Registration Monitoring System (VRMS) using personal identification information prior to this study [11] (Figure S2). The HDIS of the Beijing Municipal Health Commission Information Center covers admissions to all secondary and tertiary level hospitals in Beijing, with the exception of first-level hospitals which directly provide comprehensive medical, prevention, rehabilitation, and health care services to communities [12]. AMI admissions were identified from the Beijing HDIS based on principal discharge diagnoses with codes I21–I22 of the International Classification of Diseases, Tenth Revision (ICD-10) [13]. The VRMS of the Beijing Center for Disease Control and Prevention covers all deaths in Beijing [14]. Deaths from AMI, which included those that occurred both in and out of hospital, were identified from the Beijing VRMS according to the underlying causes of death using the ICD-10 codes (I21–I22) [13]. Information on the age, sex, onset date, and residential address of each of the AMI cases were obtained directly from the BCDSS. The diagnosis of AMI in the BCDSS has been validated as described in the method S1.

We took multiple preventative steps to avoid double counting AMI events. Cases of in-hospital deaths identified in both the Beijing HDIS and the Beijing VRMS were considered single hospitalized cases. Cases of patients who were discharged and then readmitted on the same day (including transferred patients) were deemed single continuous care episodes (*n* = 6889). Patients who were discharged following a total length of stay of ≤1 day and without readmission or death on the same day were excluded (*n* = 3533), as they were unlikely to be AMI cases [15]. Cases of hospital admission or death recorded for the same patient within a 28-day period were considered single events (*n* = 5874) in line with the World Health Organization’s monitoring trends and determinants in cardiovascular disease (WHO-MONICA) protocols [16]. Finally, a total of 259,830 AMI events among permanent Beijing residents aged ≥35 years recorded in the BCDSS between 2007 and 2018 were included in this analysis (Figure S3). The residential address of each patient who experienced an AMI event was geocoded and converted to latitude and longitude. All AMI events were geographically aggregated within townships according to the assigned latitude and longitude coordinates.

Annual population data from 2007–2018 by sex at township level were extracted from the statistical yearbooks for districts issued by the Beijing Municipal Bureau Statistics. Because population data stratified by age and sex are only reported at the district level, township population by age and sex was estimated by applying the district level population distributions. The population estimation method referred to a previous study which also used the population distributions of the larger-level spatial unit to estimate the population distributions of the smaller-level spatial unit [17].

### 2.3. Statistical Analysis

AMI incidence estimates in small areas such as townships may be unstable owing to small numbers of events. We aggregated AMI events and population into four 3 year periods (2007–2009, 2010–2012, 2013–2015, and 2016–2018) to ensure there were sufficient numbers of events for stable estimates at the township level. Then, township AMI incidences were estimated separately for males and females, each age group (35–49 years, 50–64 years, 65–79 years, and ≥80 years), and each 3 year period using the Bayesian spatial model. The model balances between overly unstable within-township estimates and overly simplified aggregate large-area estimates that mask small-area variation. The details of the model were described in the method S2.

We reported the posterior mean of AMI incidence rates by sex, age groups, and 3 year periods for each township. Age- and sex-standardized rates were calculated with weights derived from the distribution of Beijing’s population by age and sex in the 2010 national population census. We measured the absolute and relative geographic inequalities in AMI incidence rates among townships by using the difference between the 90th and the 10th percentiles of AMI incidence rates in townships and the ratio of the 90th to the 10th percentiles of AMI incidence rates in townships, respectively, according to previous literature [7].

To assess the reliability of the permanent population reporting in the statistical yearbooks, we used a Bland–Altman graph to compare district populations recorded in the 2010 national population census and the population data from statistical yearbook for the same year. All the districts except for Haidian had good consistency for the population data between the census and the statistical yearbook (Figure S4). Therefore, a sensitivity analysis was performed to assess the robustness of the results after excluding data from Haidian District.

Data management and analyses were performed using the SAS software, version 9.4 (SAS Institute Inc., Cary, NC, USA), OpenBUGS software, version 3.2.3 (MRC Biostatistics Unit, Cambridge, UK), and the ArcGIS software, version 10.5 (ESRI Inc., Redlands, CA, USA).

## 3. Results

### 3.1. Temporal Trends in AMI Incidence

A total of 259,830 fatal and non-fatal AMI events occurred among permanent residents of Beijing aged ≥35 years between 2007 and 2018, and 35.8% of patients were female. At the township level, the median age- and sex-standardized AMI incidence rate increased by 7.1% from 216.3 per 100,000 population in 2007–2009 to 231.6 per 100,000 population in 2016–2018. The median age-standardized rate increased by 14.7% for males, from 288.6 to 331.1 per 100,000 population, whereas the rate for females decreased by 8.1%, from 145.9 to 134.1 per 100,000 population during the study period. Further stratified analyses by sex and age groups revealed distinct trends. The most pronounced increases in the median incidence rate were observed among young and middle-aged males (35–49 years: 54.2%; 50–64 years: 33.2%), whereas those for other age–sex groups ranged between −15.1% and 1.5% (Table 1).

### 3.2. Geographic Pattern and Inequality in AMI Incidence

**Figure 1:**
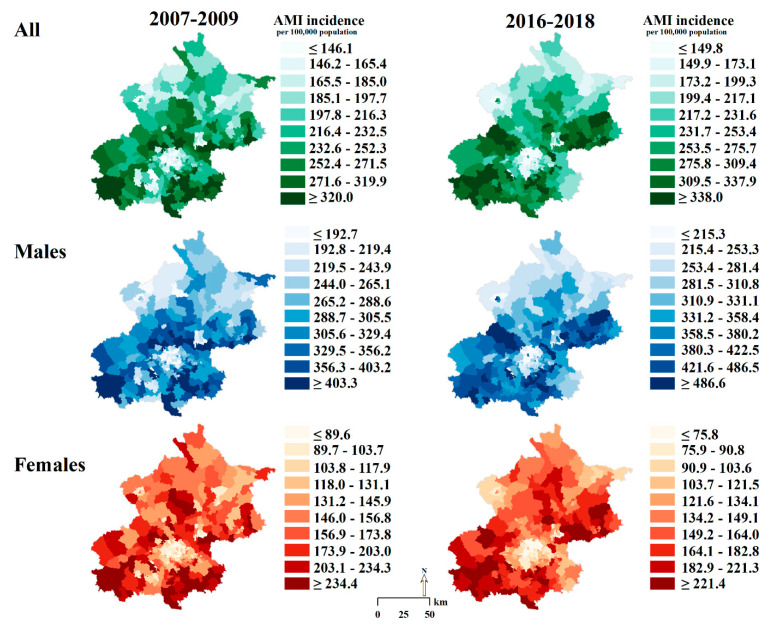
Deciles of the Age-Standardized Incidence of Acute Myocardial Infarction in Beijing Residents Aged ≥35 Years at the Township Level, 2007–2018. AMI indicates acute myocardial infarction.

**Figure 2:**
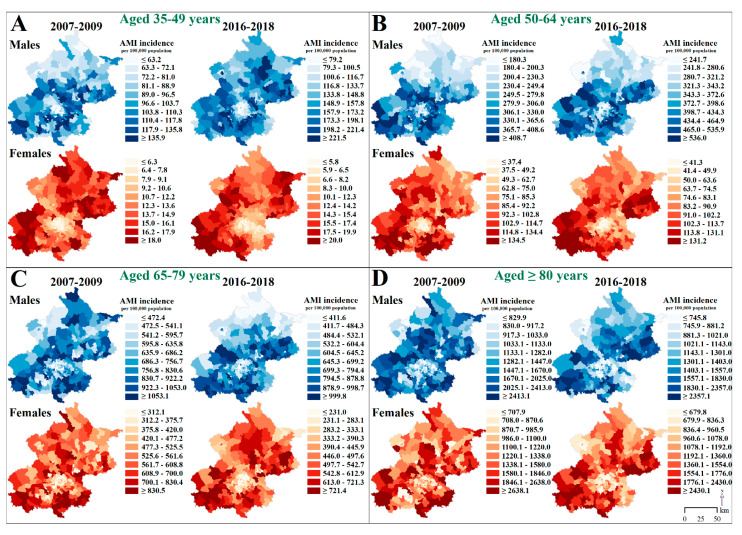
Deciles of the Incidence of Acute Myocardial Infarction by Age–Sex Groups at the Township Level in Beijing, 2007–2018. AMI indicates acute myocardial infarction.

Townships with high AMI rates (in the top decile) were mainly found in the southern peri-urban areas of Beijing in 2007–2009. In 2016–2018, townships with high AMI rates emerged in the northeastern peri-urban areas and highly clustered in the southwestern peri-urban areas, while the high-rate townships in the southeastern peri-urban areas had dissipated (Figure 1). Townships with high AMI rates among young males aged 35–49 years were more clustered in the peri-urban areas surrounding the urban core by 2016–2018. For other age–sex groups, the geographic patterns in AMI incidence were similar to those of the general population during the study period (Figure 2).

Overall, the absolute geographic inequality, which was defined as the difference between the 90th and 10th percentile of the AMI incidence rate in townships, increased slightly from 173.8 per 100,000 population in 2007–2009 to 188.1 per 100,000 population in 2016–2018. Moreover, the relative geographic inequality, defined as the ratio of the 90th to the 10th percentile of the AMI incidence rate in townships, were 2.2 and 2.3 in 2007–2009 and 2016–2018, respectively. The increase in relative geographic inequality was more apparent in the case of young residents aged 35–49 years (2.1 to 2.8 for males and 2.8 to 3.4 for females) compared with older residents aged 65–79 years (2.2 to 2.4 for males and 2.7 to 3.1 for females) and those aged ≥80 years (2.9 to 3.2 for males and 3.7 to 3.6 for females) (Table 2).

### 3.3. Geographic Patterns in Changes of AMI Incidence

**Figure 3:**
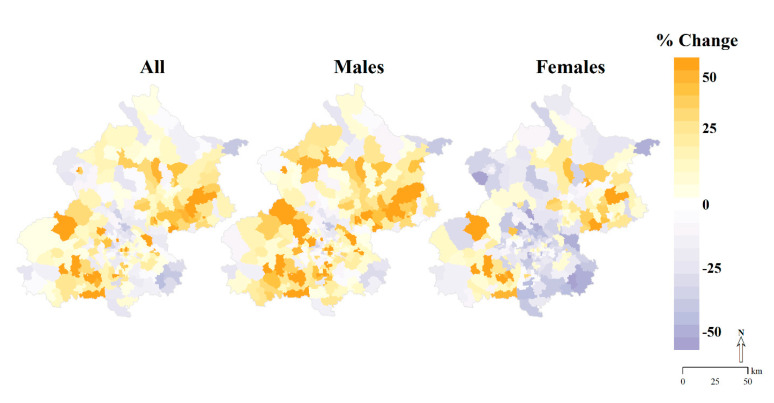
Percentage Changes in the Age−Standardized Incidence of Acute Myocardial Infarction in Beijing Residents Aged ≥35 Years at the Township Level, 2007–2018.

**Figure 4:**
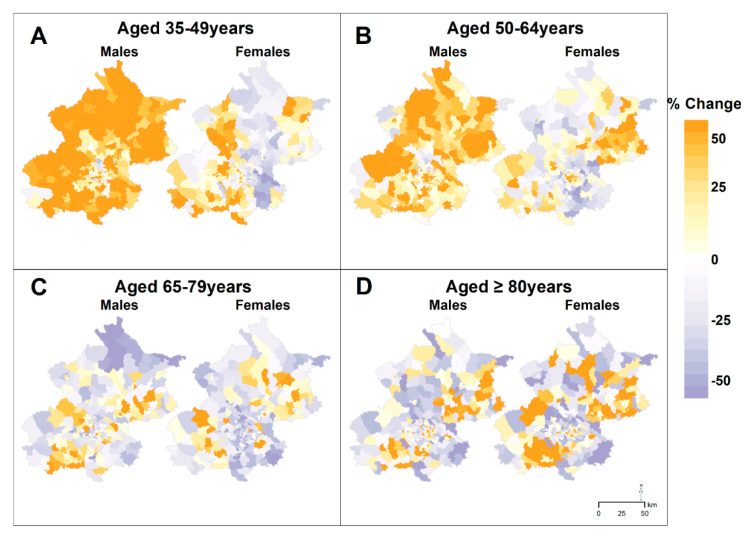
Percentage Changes in the Incidence of Acute Myocardial Infarction by Age−Sex Groups at the Township Level in Beijing, 2007–2018.

Most townships with the increase in AMI incidence by 50% or greater were located in the southwestern and northeastern peri-urban areas of Beijing, and geographic patterns were similar for males and females. Townships with large declines in incidences of AMI among females were clustered in the southeastern of Beijing (Figure 3). We found increases in AMI incidence rates of 50% or greater for young and middle-aged males aged 35–64 years in almost all peri-urban areas. Declining incidence rates for AMI among other age-sex groups were mainly found in townships located in the southeastern of Beijing (Figure 4).

### 3.4. Sensitivity Analyses

We obtained similar results after excluding Haidian District from the analysis due to inconsistencies in the recorded population, although overall geographic differences were smaller (Tables S1 and S2; Figures S5–S8).

## 4. Discussion

This study reveals the temporal trends in the AMI incidence at the township level in Beijing over a decade using the most recent data and shows high-resolution inequalities in the AMI incidence within a global city located in a developing country. We observed an increase in the median AMI incidence rate in males, particularly those who were young and middle-aged, and, conversely, a decline in the AMI incidence among older adults during the study period. High rates and rapid increases in the AMI incidence were particularly evident in the city’ s southwestern and northeastern peri-urban areas. Moreover, geographic inequalities in the AMI incidence across townships increased dramatically among younger residents over the 12 year period of observation.

Our findings show substantial geographic inequalities in the incidence of AMI at the township level in Beijing. In a previous study conducted at the district level in Beijing, hotspots of admissions for coronary heart disease were observed in the southwestern peri-urban Fangshan District [18]. We found similar high-risk areas for AMI incidence. However, our study, which was conducted at a smaller spatial scale, revealed within-district variation that may be masked by the district-level averages and expanded the evidence for implementing targeted actions focusing on specific high-risk areas and populations.

The temporal trends and geographic patterns of AMI incidence observed in our study may be partly attributed to the changes and variations in the prevalence, awareness, and control rates of CVD risk factors in Beijing. The increasing prevalence of diabetes and hypertension within Beijing’ s population may contribute to the increased incidence of AMI among young and middle-aged males. Studies have shown that the prevalence of diabetes among Beijing residents aged <40 years increased annually by 13.6% from 2008 to 2017 [19], while the prevalence of hypertension among residents aged 45–54 years increased by 5.4% from 2005 to 2011 [20]. Additionally, awareness and control rates of CVD risk factors are lower among young and middle-aged residents compared with older residents [20,21], who are more likely to visit healthcare services [22]. The decline in AMI incidence among older residents in our study may be partly attributable to the improved risk factor management. For example, from 2005 to 2011, awareness and control rate of hypertension among older residents in Beijing increased by 11.4% and 10.8%, respectively [20]. Geographically, the concentration of townships with high and increased incidence of AMI in the southwestern and northeastern peri-urban areas may be attributed to the higher prevalence of CVD risk factors in these areas. Recent studies conducted in Beijing found a higher prevalence of CVD risk factors (e.g., diabetes, hypertension, and higher body mass index) in the districts of Fangshan (in the southwestern) and Pinggu (in the northeastern) compared with the average prevalence among residents of Beijing as a whole [19,20,23,24,25,26].

Furthermore, there are a number of local public health policies that may explain some of the temporal trends and geographic patterns of AMI incidence observed in our study. First, a series of tobacco control policies implemented in Beijing over the last decade may play a role in the declining trend of AMI incidence among older residents. In 2008, Beijing enforced a smoking ban in 11 types of public places [27]. One study found that, between 2001 and 2010, the prevalence of smoking declined by 3.5% (from 24.7% to 21.2%) among urban older males and nearly halved (declining from 8.8% to 4.1%) among urban older females [28]. In addition, a previous study found that a significant reduction in ischemic heart disease was observed only among older residents of Beijing and not among young and middle-aged residents after the launch of the Beijing Municipal Tobacco Control Regulation and the implementation of the National Tobacco Tax Reform in 2015 [29]. Second, Tongzhou District, which has been the location of the Beijing Municipal Administrative Center since 2012, is vigorously promoting the improvement of medical resources, and ranks the highest among Beijing’s districts for its increase in medical staff (160% between 2007 and 2018) [30]. This could partially contribute to the decrease in the AMI incidence in the southeastern part of Beijing where Tongzhou District is located. Third, to improve the air quality, the government has enforced a series of policies and control measures in Beijing, notably the “Regulations of Beijing Municipality on Atmospheric Pollution Prevention and Control” [31] and the “Beijing 2013–2017 Clean Air Action Plan” [32]. Although PM_2.5_ (particulate matter) concentrations decreased in most areas of Beijing between 2013 and 2018, townships with high incidence of AMI located in the southwestern districts, such as Fangshan, continued to rank the highest for air pollution compared with other districts in Beijing [33], which may have partly contributed to the inequalities in AMI incidence.

The findings of our study advance the understanding of regional health disparities and have several implications for effective CVD control. First, our small-area study at the township level provides targeted guidance on priority areas for preventive activities, given that a township is the smallest official administrative unit that plays a crucial role in health-related resource allocation and the implementation of prevention and treatment programs in China. Second, community-based screening programs, effective at improving CVD risk factors [34], are essential for CVD control. Third, interventions are needed to improve accessibility to health care services and ensure adequate medications, and low-cost guideline-recommended medications should be prioritized in primary health-care settings [35]. These recommendations are particularly important for developing countries where there is usually an imbalance between a large and growing CVD burden [2] and relatively limited allocation of resources for treating non-communicable diseases [36]. Last, we found that temporal trends and geographic patterns in AMI incidence coincided with the implementation of certain public policies, such as tobacco control, improvement of medical resources, and air pollution control. Therefore, more rigorous further studies focusing on evaluating the impacts of these policies on AMI are warranted, which may be helpful for the implementation of effective policies targeting preventing CVD and reducing health inequalities.

Our study had several notable strengths. First, data analysis in most previous geographic studies has focused either on AMI hospital admissions or mortality, which may lead to underestimation of the total AMI burden [37,38,39]. We obtained data on AMI incidence that covered both hospitalized AMI cases and out-of-hospital deaths from the BCDSS [12,14], thus providing more accurate depictions of spatiotemporal patterns for the AMI burden. In addition, by borrowing statistical strength across space (neighboring townships), our Bayesian model produced more robust estimates of AMI incidence at the small area level [40].

Our study has several limitations to be considered for future studies. First, the total population of Haidian District recorded in the statistical yearbook was larger than that recorded in the population census conducted in 2010, which could lead to an underestimation of the overall AMI incidence in Haidian District. However, the results of the sensitivity analysis, in which Haidian District was excluded, supported our main findings, although geographic inequalities were smaller than the main results. Second, we were unable to examine the relative contribution of potential influencing factors to regional disparities in AMI incidence due to the unavailability of associated factor data at township level in Beijing. Further association studies focusing on the potential factors and policies that influence AMI are warranted to better understand the inequities of AMI incidence. Moreover, this observational retrospective study is limited to a Beijing population, whereas the methodology and findings from this study might be helpful for further studies on trends and inequalities in AMI incidence in other megacities which experience rapid development as that of Beijing.

## 5. Conclusions

Substantial disparities exist in the incidence of AMI at the township level in Beijing, with dramatic increases observed among young and middle-aged males from 2007 to 2018. Hotspots and rapid increases in AMI incidence occurred in the southwestern and northeastern peri-urban areas of the city, and geographic inequality in AMI incidence increased among younger residents. Our findings highlight the need for more targeted actions aimed at AMI prevention and control among high-risk populations and areas to reduce both the overall cardiovascular burden in Beijing and regional disparities.

## Figures and Tables

**Table 1:** The Incidence of Acute Myocardial Infarction by Township, 2007–2018 (per 100,000 population).

Characteristic	2007–2009		2010–2012		2013–2015		2016–2018	
	Median	IQR	Median	IQR	Median	IQR	Median	IQR
Total ^a^	216.3	(176.0, 259.5)	239.5	(192.9, 289.4)	236.2	(184.5, 297.3)	231.6	(188.4, 286.1)
Males ^b^	288.6	(230.1, 337.6)	316.4	(253.1, 376.7)	318.9	(253.7, 393.2)	331.1	(267.5, 396.9)
35–49 years	96.5	(76.7, 113.9)	118.8	(95.4, 142.6)	137.0	(103.7, 170.9)	148.8	(108.6, 183.4)
50–64 years	279.8	(215.2, 342.8)	317.5	(258.3, 378.2)	336.1	(264.8, 416.7)	372.6	(297.6, 447.6)
65–79 years	686.2	(577.7, 855.5)	729.6	(577.7, 911.4)	669.3	(541.5, 894.8)	645.2	(506.0, 831.5)
≥80 years	1282.0	(976.1, 1852.0)	1378.0	(1021.0, 1832.0)	1262.0	(958.8, 1733.0)	1301.0	(970.0, 1718.0)
Females ^b^	145.9	(108.5, 186.1)	162.8	(116.6, 206.6)	148.2	(106.5, 199.4)	134.1	(98.3, 174.3)
35–49 years	12.2	(8.2, 15.7)	12.3	(7.5, 16.3)	12.6	(8.0, 18.4)	12.3	(7.2, 16.0)
50–64 years	85.3	(37.4, 134.4)	100.3	(63.6, 124.5)	87.2	(54.4, 116.8)	83.1	(57.3, 105.8)
65–79 years	525.5	(393.0, 639.3)	562.9	(421.2, 695.6)	500.6	(370.7, 657.5)	445.9	(315.8, 574.5)
≥80 years	1220.0	(917.2, 1670.0)	1372.0	(940.0, 1974.0)	1317.0	(937.9, 1883.0)	1192.0	(881.4, 1687.0)

^a^ Age- and sex-standardization according to the population distribution of the 2010 Beijing population census. ^b^ Age-standardization according to the population distribution of the 2010 Beijing population census. IQR indicates interquartile range.

**Table 2:** Inequalities in the Incidence of Acute Myocardial Infarction by Township, 2007–2018 (per 100,000 population).

	2007–2009				2010–2012				2013–2015				2016–2018			
Characteristic	10th Percentile	90th Percentile	90th–10th ^a^	90th/ 10th ^b^	10th Percentile	90th Percentile	90th–10th ^a^	90th/ 10th ^b^	10th Percentile	90th Percentile	90th–10th ^a^	90th/ 10th ^b^	10th Percentile	90th Percentile	90th–10th ^a^	90th/ 10th ^b^
Total ^c^	146.1	319.9	173.8	2.2	147.5	352.1	204.6	2.4	147.5	355.5	208.0	2.4	149.8	337.9	188.1	2.3
Males ^d^	192.7	403.2	210.5	2.1	205.1	446.1	241.0	2.2	211.8	470.3	258.5	2.2	215.3	486.5	271.2	2.3
35–49 years	63.2	135.8	72.6	2.1	77.9	159.3	81.4	2.0	82.6	203.1	120.5	2.5	79.2	221.4	142.2	2.8
50–64 years	180.3	408.6	228.3	2.3	203.5	433.1	229.6	2.1	202.8	482.6	279.8	2.4	241.7	535.9	294.2	2.2
65–79 years	472.4	1053.0	580.6	2.2	458.7	1193.0	734.3	2.6	456.4	1087.0	630.6	2.4	411.6	998.7	587.1	2.4
≥ 80 years	829.9	2413.0	1583.1	2.9	798.6	2589.0	1790.4	3.2	693.1	2425.0	1731.9	3.5	745.8	2357.0	1611.2	3.2
Females ^d^	89.6	234.3	144.7	2.6	85.5	262.5	177.0	3.1	80.8	248.8	168.0	3.1	75.8	221.3	145.5	2.9
35–49 years	6.3	17.9	11.6	2.8	6.0	22.1	16.1	3.7	5.8	27.2	21.4	4.7	5.8	19.9	14.1	3.4
50–64 years	37.4	134.4	97.0	3.6	38.9	153.3	114.4	3.9	37.2	147.7	110.5	4.0	41.3	131.1	89.8	3.2
65–79 years	312.1	830.4	518.3	2.7	316.3	932.4	616.1	2.9	291.6	855.3	563.7	2.9	231.0	721.3	490.3	3.1
≥80 years	707.9	2638.0	1930.1	3.7	723.4	3042.0	2318.6	4.2	705.5	2655.0	1949.5	3.8	679.8	2430.0	1750.2	3.6

^a^ Difference between the 90th and the 10th percentiles of AMI incidence rate in townships, as a measure of absolute geographic inequality. ^b^ Ratio of the 90th to the 10th percentiles of AMI incidence rate in townships, as a measure of relative geographic inequality. ^c^ Age- and sex-standardization according to the population distribution of the 2010 Beijing population census. ^d^ Age-standardization according to the population distribution of the 2010 Beijing population census.

## Data Availability

The data used for this study were obtained from the Beijing Municipal Health Commission Information Center and cannot be shared publicly, given their institutional regulations and the data confidentiality agreement. Similar data may be requested by researchers from the above data holder authorities for research purposes. The analytical methods can be reproduced based on the details provided in this article, and the statistical code is available upon request.

## Supplementary Materials

The following are available online at https://www.mdpi.com/article/10.3390/ijerph182312276/s1, Method S1: The validation of the diagnosis of acute myocardial infarction in the Beijing Cardiovascular Disease Surveillance System. Method S2: The Bayesian spatial model. Table S1: The Incidence of Acute Myocardial Infarction in Beijing Residents Aged ≥35 Years at the Township Level after Excluding Data from Haidian District, 2007–2018 (per 100,000 population). Table S2: Inequalities in the Incidence of Acute Myocardial Infarction in Beijing Residents Aged ≥35 Years at the Township Level after Excluding Data from Haidian District (per 100,000 population). Figure S1: Townships in Urban Cores and Peri-urban Areas in Beijing. Figure S2: Data Source and Linkage of the Beijing Cardiovascular Disease Surveillance System. Figure S3: Flowchart of the Study Population. Figure S4: The Bland–Altman Plot of Permanent Population by Districts from Census and Yearbook in the Same Year. Figure S5: Deciles of the Age-Standardized Incidence of Acute Myocardial Infarction in Beijing Residents Aged ≥35 Years at the Township Level after Excluding Data from Haidian District, 2007–2018. Figure S6: Deciles of the Incidence of Acute Myocardial Infarction by Age-Sex Groups at the Township Level in Beijing after Excluding Data from Haidian District, 2007–2018. Figure S7: Percentage Changes in the Age-Standardized Incidence of Acute Myocardial Infarction in Beijing Residents Aged ≥35 Years at the Township Level after Excluding Data from Haidian District, 2007–2018. Figure S8: Percentage Changes in the Incidence of Acute Myocardial Infarction by Age-Sex Groups at the Township Level in Beijing after Excluding Data from Haidian District, 2007–2018. References [41,42] are cited in the supplementary materials.

## Abbreviations

AMI: acute myocardial infarction; CVD: cardiovascular disease; BCDSS: Beijing cardiovascular disease surveillance system; HDIS: hospital discharge information system; VRMS: vital registration monitoring system; ICD-10: international classification of diseases, tenth revision; WHO-MONICA: World Health Organization’s monitoring trends and determinants in cardiovascular disease.
