# Supplementary material for: Trends and Inequalities in the Incidence of Acute Myocardial Infarction among Beijing Townships, 2007–2018
Source: Int J Environ Res Public Health. 2021 Nov 23;18(23):12276. doi: 10.3390/ijerph182312276 (PMC8656834; doi:10.3390/ijerph182312276)
Supplement: Supplementary file 1 [file ijerph-18-12276-s001.zip › ijerph-1442050-supplementary.pdf]

SUPPLEMENTAL MATERIAL

Trends and Inequalities in the Incidence of Acute Myocardial Infarction among Beijing Townships, 2007–2018

Supplemental Methods

Method S1. The validation of the diagnosis of acute myocardial infarction in the Beijing Cardiovascular Disease Surveillance System

The diagnoses in the Beijing Cardiovascular Disease Surveillance System have been previously validated, with an "almost perfect" or "substantial" agreement (Kappa statistic range: 0.725 to 0.880) between the ICD code-based diagnoses in the system and clinical diagnoses in the original hospital charts. The positive predictive values of acute myocardial infarction diagnosis in the system were 94.4% and 87.9% when compared with the criteria of the World Health Organization's Monitoring Trends and Determinants in Cardiovascular Disease (WHO-MONICA) project and the Third Universal Definition of acute myocardial infarction, respectively. The corresponding negative predictive values were 96.1% and 97.0%, respectively [41].

Method S2. The Bayesian spatial model

The model was expressed as follows:

$$Events_d \sim Poisson(m_d.Population_d)$$

$$log(m_d) = \alpha_0 + b_d + h_d$$

where $Events_d$, $Population_d$, and m_d are the number of events, populations, and estimate rates, respectively, for township d for each period-sex-age group. The α_0 is the common intercept. The terms *b* and *h* are components of the Besag-York-Mollié (BYM) model and are described in detail elsewhere [42]. Briefly, the random effects denoted by *b* were assumed to follow conditional autoregressive distributions that allowed for smoothing over adjacent townships' rates. The random effects denoted by *h* were assumed to follow independent mean zero normal distributions. In the BYM model, the estimated AMI incidence rate in each township was influenced by its own data and by the data of neighboring townships through spatially structured random effects with conditional autoregressive prior distribution as well as globally through a spatially unstructured normal prior distribution.

Supplemental Tables

Table S1. The Incidence of Acute Myocardial Infarction in Beijing Residents Aged ≥ 35 Years at the Township Level after Excluding Data from Haidian District, 2007–2018 (per 100,000 population)

	2007–2009		2010–2012		2013–2015		2016–2018	
	Median	IQR	Median	IQR	Median	IQR	Median	IQR
Total [a]	222.1	(186.5, 261.9)	245.4	(208.3, 295.4)	240.2	(199.9, 297.8)	239.7	(201.5, 286.7)
Males [b]	291.7	(241.0, 339.2)	326.8	(270.2, 381.5)	325.5	(268.4, 394.5)	336.2	(281.2, 404.5)
35–49 years	100.1	(82.2, 115.3)	123.8	(102.9, 142.9)	141.5	(114.8, 171.7)	152.9	(119.3, 188.8)
50–64 years	283.8	(220.1, 347.9)	322.2	(270.8, 383.3)	343.3	(279.5, 415.5)	377.4	(317.9, 446.5)
65–79 years	713.9	(590.0, 865.5)	747.6	(608.7, 918.1)	684.5	(572.3, 896.8)	659.1	(533.5, 822.1)
≥ 80 years	1313.0	(1005.0, 1869.0)	1408.8	(1083.0, 1871.0)	1301.0	(1029.0, 1759.0)	1336.0	(1009.0, 1764.0)
Females [b]	150.6	(118.3, 187.1)	167.8	(128.1, 210.2)	155.6	(116.4, 202.9)	135.9	(104.3, 175.5)
35–49 years	12.9	(9.9, 15.4)	13.2	(8.4, 17.0)	13.8	(9.7, 19.5)	13.2	(7.9, 16.2)
50–64 years	88.4	(62.3, 108.5)	104.3	(72.8, 127.3)	90.6	(63.7, 118.2)	86.9	(65.2, 105.8)
65–79 years	543.0	(424.7, 650.7)	580.0	(465.4, 715.6)	518.7	(402.2, 666.5)	468.1	(332.8, 583.0)
≥ 80 years	1250.0	(937.7, 1718.0)	1449.0	(992.8, 2038.0)	1392.0	(1004.0, 1948.0)	1232.0	(954.1, 1723.0)

[a] Age- and sex-standardization according to the population distribution of the 2010 Beijing population census.

[b] Age-standardization according to the population distribution of the 2010 Beijing population census.

IQR indicates interquartile range.

Table S2. Inequalities in the Incidence of Acute Myocardial Infarction in Beijing Residents Aged ≥ 35 Years at the Township Level after Excluding Data from Haidian District (per 100,000 population)

	2007–2009				2010–2012				2013–2015				2016–2018			
	10th percentile	90th percentile	90th - 10th [a]	90th/ 10th [b]	10th percentile	90th percentile	90th - 10th [a]	90th/ 10th [b]	10th percentile	90th percentile	90th - 10th [a]	90th/ 10th [b]	10th percentile	90th percentile	90th - 10th [a]	90th/ 10th [b]
Total [c]	155.0	319.0	164.0	2.1	165.4	355.8	190.4	2.2	165.7	353.5	187.8	2.1	165.8	343.7	177.9	2.1
Males [d]	201.2	402.0	200.8	2.0	229.2	445.4	216.2	1.9	228.9	466.7	237.8	2.0	235.9	481.2	245.3	2.0
35–49 years	70.3	138.3	68.0	2.0	89.2	156.9	67.7	1.8	92.5	200.0	107.5	2.2	90.5	222.4	131.9	2.5
50–64 years	182.5	410.4	227.9	2.2	226.7	432.2	205.5	1.9	229.2	475.1	245.9	2.1	262.9	532.0	269.1	2.0
65–79 years	495.6	1062.0	566.4	2.1	503.2	1206.0	702.8	2.4	492.3	1079.0	586.7	2.2	447.2	969.7	522.5	2.2
≥ 80 years	833.0	2407.0	1574.0	2.9	816.4	2626.0	1809.6	3.2	727.6	2446.0	1718.4	3.4	796.3	2510.0	1713.7	3.2
Females [d]	95.8	234.1	138.3	2.4	97.6	267.2	169.6	2.7	93.5	254.7	161.2	2.7	82.0	221.4	139.4	2.7
35–49 years	7.4	16.8	9.4	2.3	6.7	22.6	15.9	3.4	6.9	26.5	19.6	3.8	6.3	19.1	12.8	3.0
50–64 years	39.0	135.1	93.1	3.5	45.2	155.0	109.8	3.4	45.6	146.4	100.8	3.2	48.5	129.2	80.7	2.7
65–79 years	338.8	837.1	498.3	2.5	356.3	931.5	575.2	2.6	329.7	848.6	518.9	2.6	255.5	719.6	464.1	2.8
≥ 80 years	706.1	2633.0	1926.9	3.7	780.2	3171.0	2390.8	4.1	797.3	2685.0	1887.7	3.4	788.0	2495.0	1707.0	3.2

[a] Difference between the 90th and the 10th percentiles of AMI incidence rate in townships, as a measure of absolute geographic inequality.

[b] Ratio of the 90th to the 10th percentiles of AMI incidence rate in townships, as a measure of relative geographic inequality.

[c] Age- and sex-standardization according to the population distribution of the 2010 Beijing population census.

[d] Age-standardization according to the population distribution of the 2010 Beijing population census.

Figure Legends

1. **Figure S1.** Townships in Urban Cores and Peri-urban Areas in Beijing
2. **Figure S2.** Data Source and Linkage of the Beijing Cardiovascular Disease Surveillance System
3. **Figure S3.** Flowchart of the Study Population

   AMI indicates acute myocardial infarction
4. **Figure S4.** The Bland-Altman Plot of Permanent Population by Districts from Census and Yearbook in the Same Year

   The diamond in the figure depict the Haidian District. All the districts except for Haidian had good consistency for the population data between the census and the statistical yearbook.
5. **Figure S5.** Deciles of the Age-Standardized Incidence of Acute Myocardial Infarction in Beijing Residents Aged ≥ 35 Years at the Township Level after Excluding Data from Haidian District, 2007–2018

   AMI indicates acute myocardial infarction
6. **Figure S6.** Deciles of the Incidence of Acute Myocardial Infarction by Age-Sex Groups at the Township Level in Beijing after Excluding Data from Haidian District, 2007–2018

   AMI indicates acute myocardial infarction.
7. **Figure S7.** Percentage Changes in the Age-Standardized Incidence of Acute Myocardial Infarction in Beijing Residents Aged ≥ 35 Years at the Township Level after Excluding Data from Haidian District, 2007–2018
8. **Figure S8.** Percentage Changes in the Incidence of Acute Myocardial Infarction by Age-Sex Groups at the Township Level in Beijing after Excluding Data from Haidian District, 2007–2018

Supplemental Figures

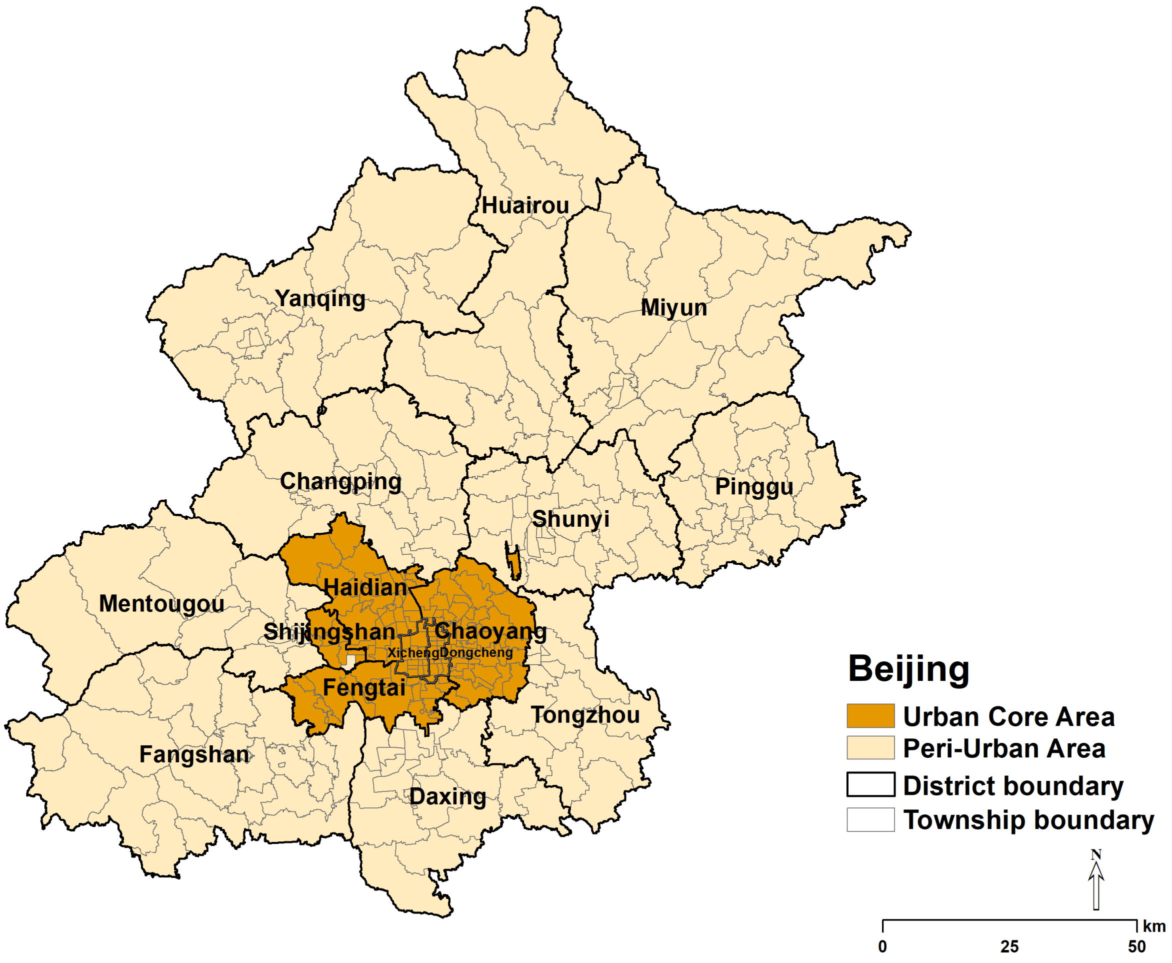

Figure S1. Townships in Urban Cores and Peri-urban Areas in Beijing

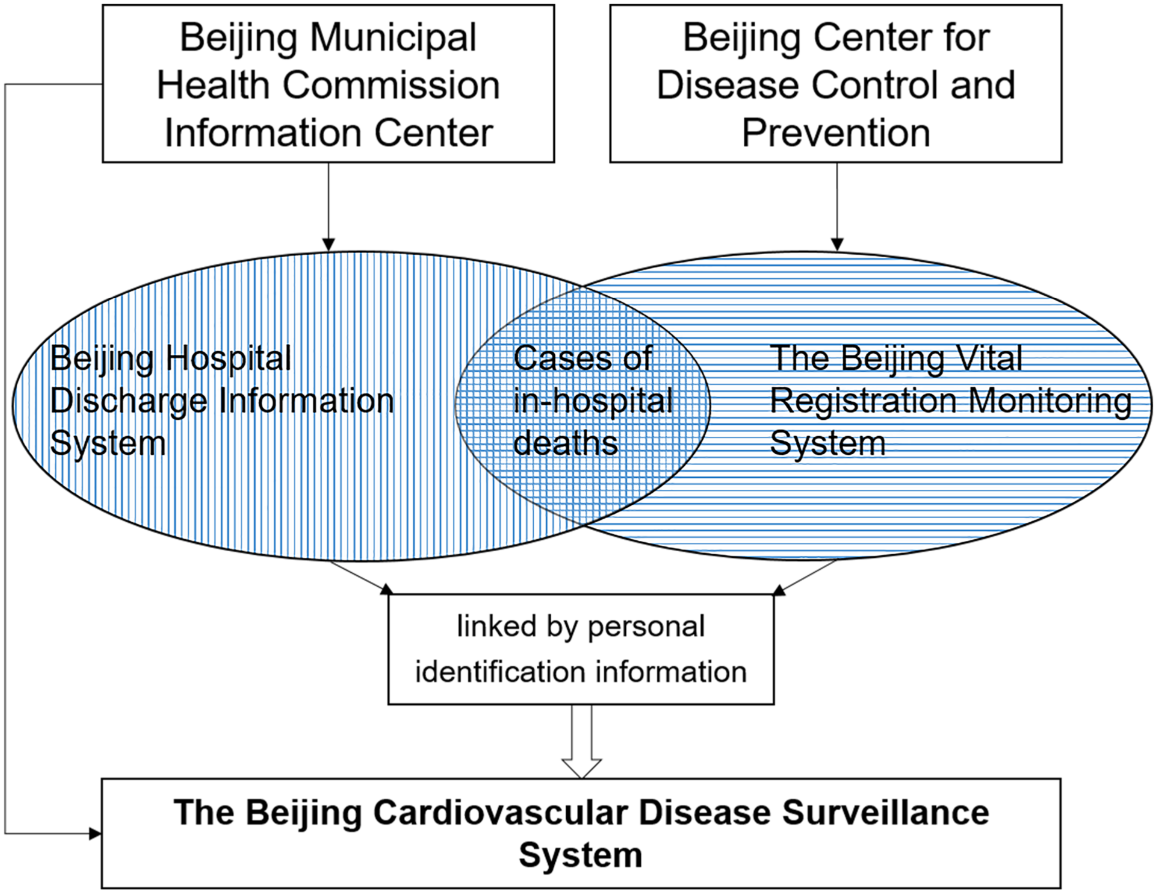

Figure S2. Data Source and Linkage of the Beijing Cardiovascular Disease Surveillance System

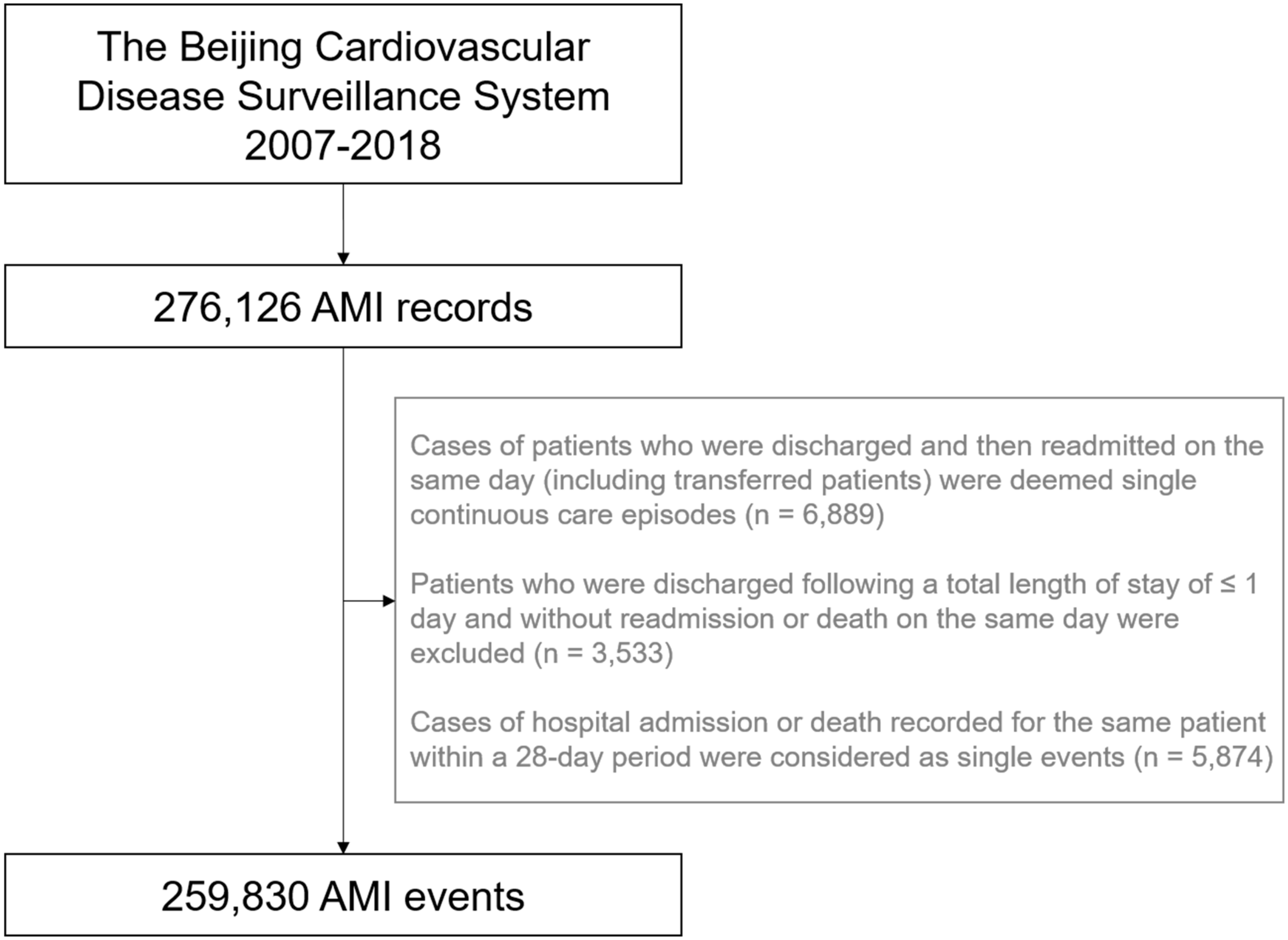

Figure S3. Flowchart of the Study Population

AMI indicates acute myocardial infarction.

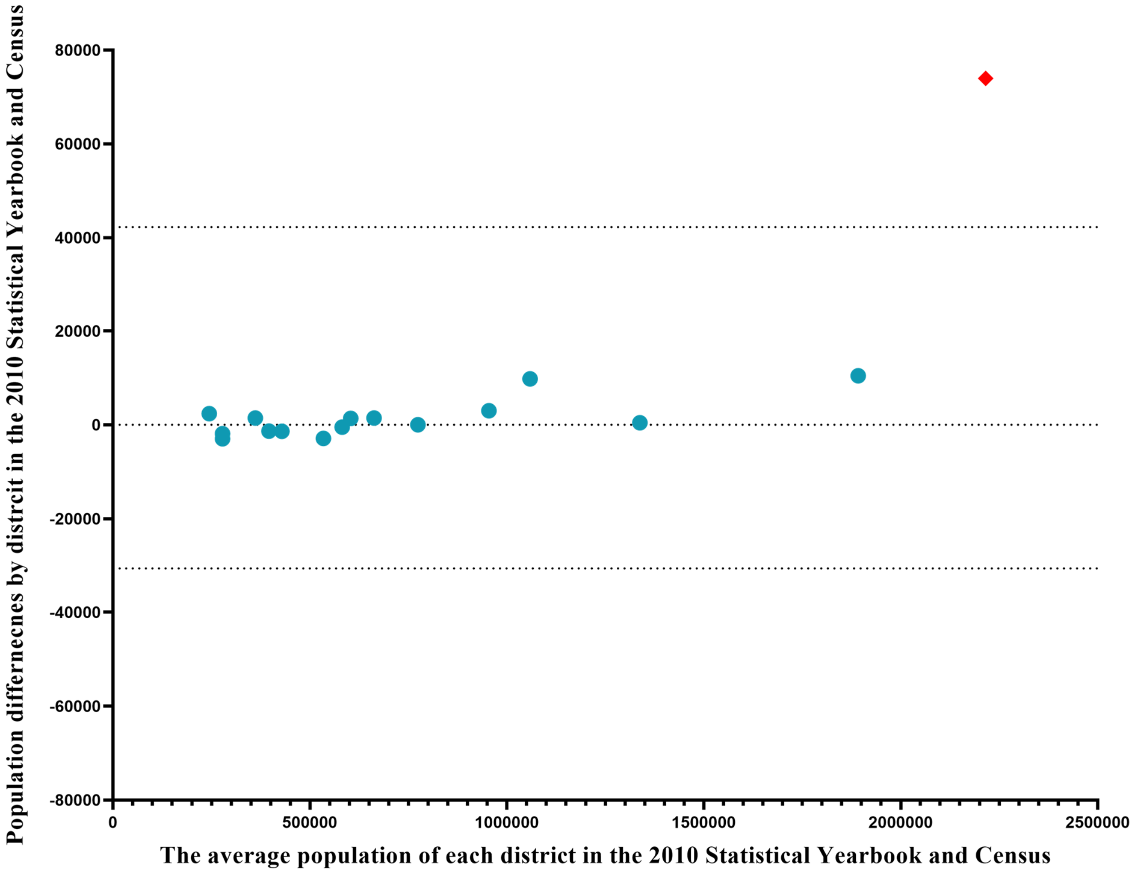

Figure S4. The Bland-Altman Plot of Permanent Population by Districts from Census and Yearbook in the Same Year

The diamond in the figure depict the Haidian District. All the districts except for Haidian had good consistency for the population data between the census and the statistical yearbook.

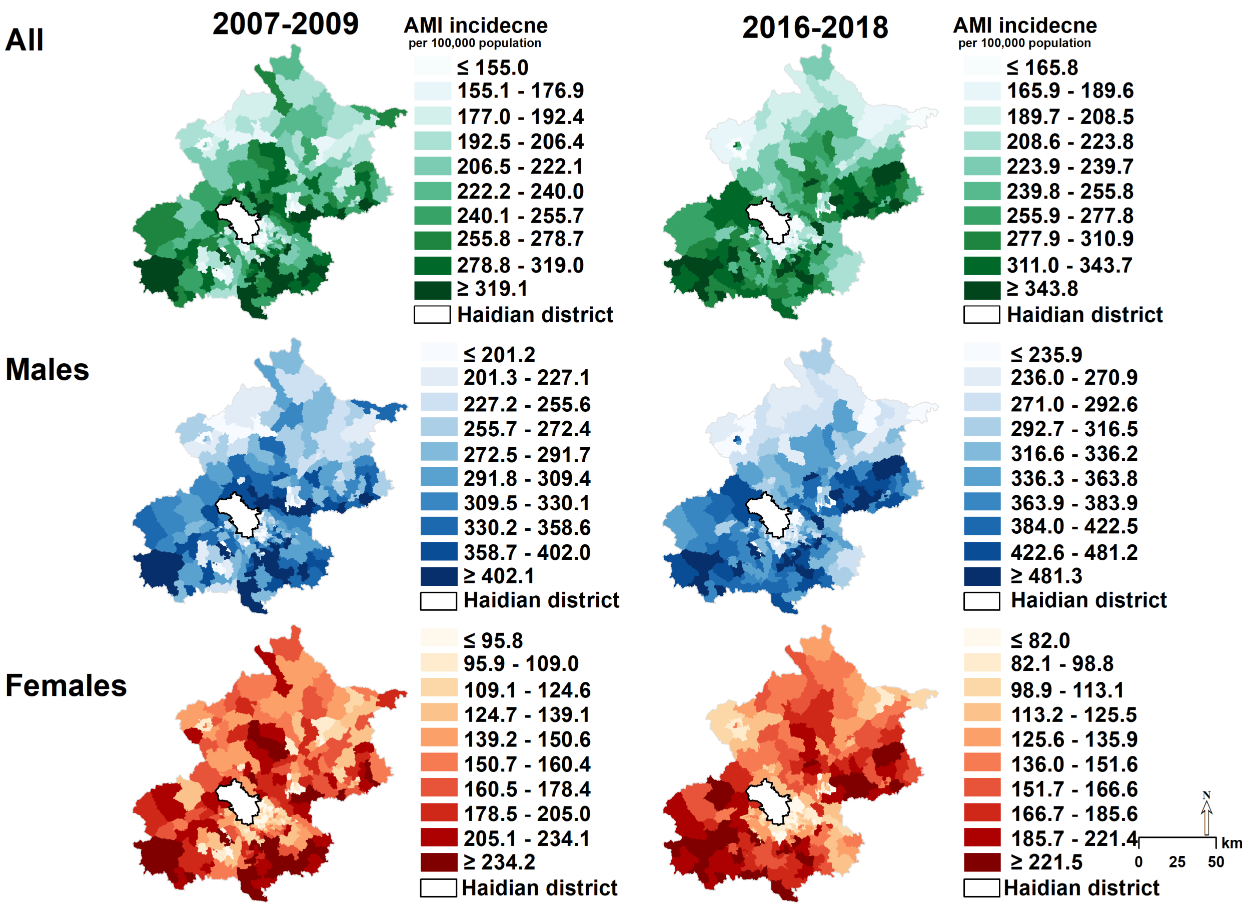

Figure S5. Deciles of the Age-Standardized Incidence of Acute Myocardial Infarction in Beijing Residents Aged ≥ 35 Years at the Township Level after Excluding Data from Haidian District, 2007–2018

AMI indicates acute myocardial infarction.

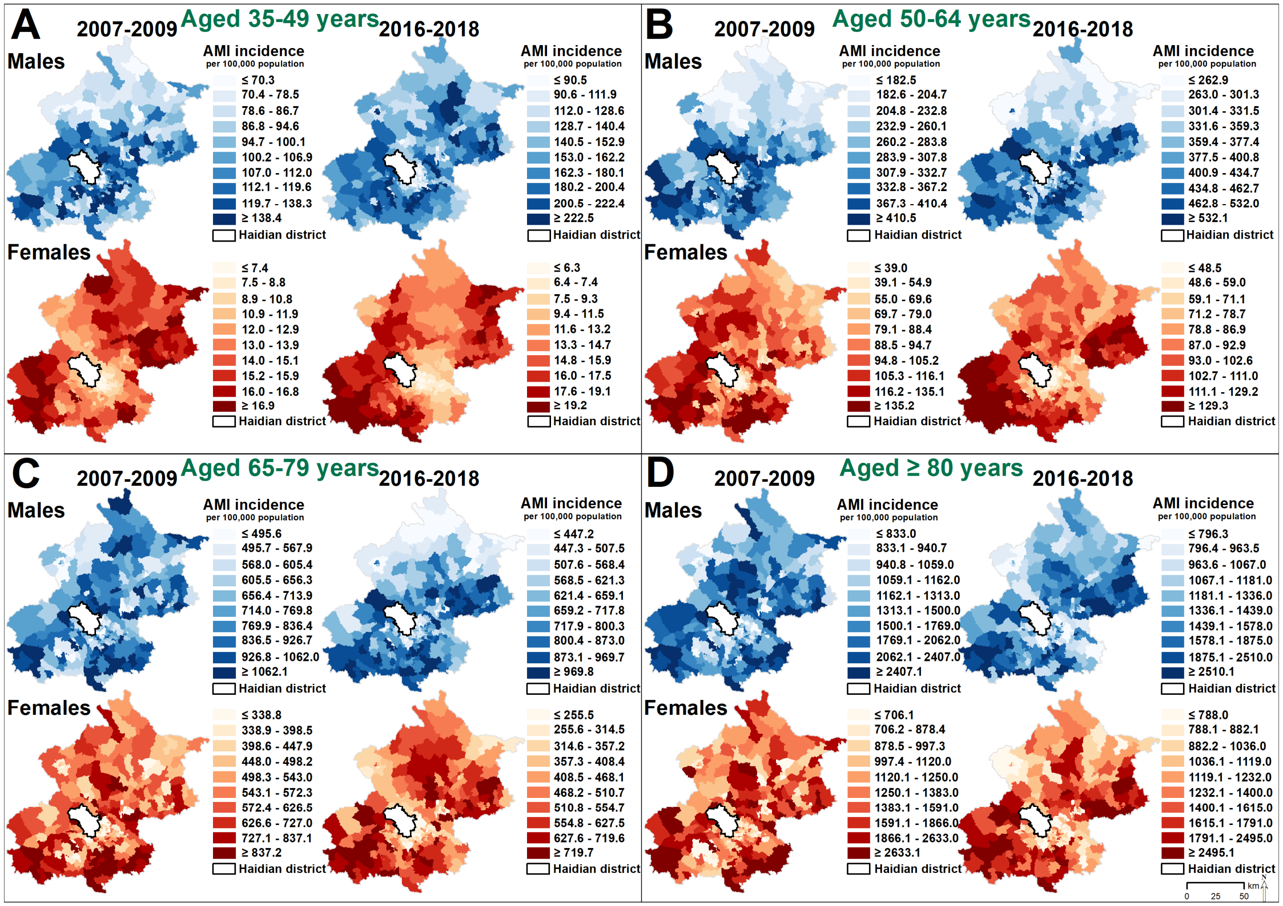

Figure S6. Deciles of the Incidence of Acute Myocardial Infarction by Age-Sex Groups at the Township Level in Beijing after Excluding Data from Haidian District, 2007–2018

AMI indicates acute myocardial infarction.

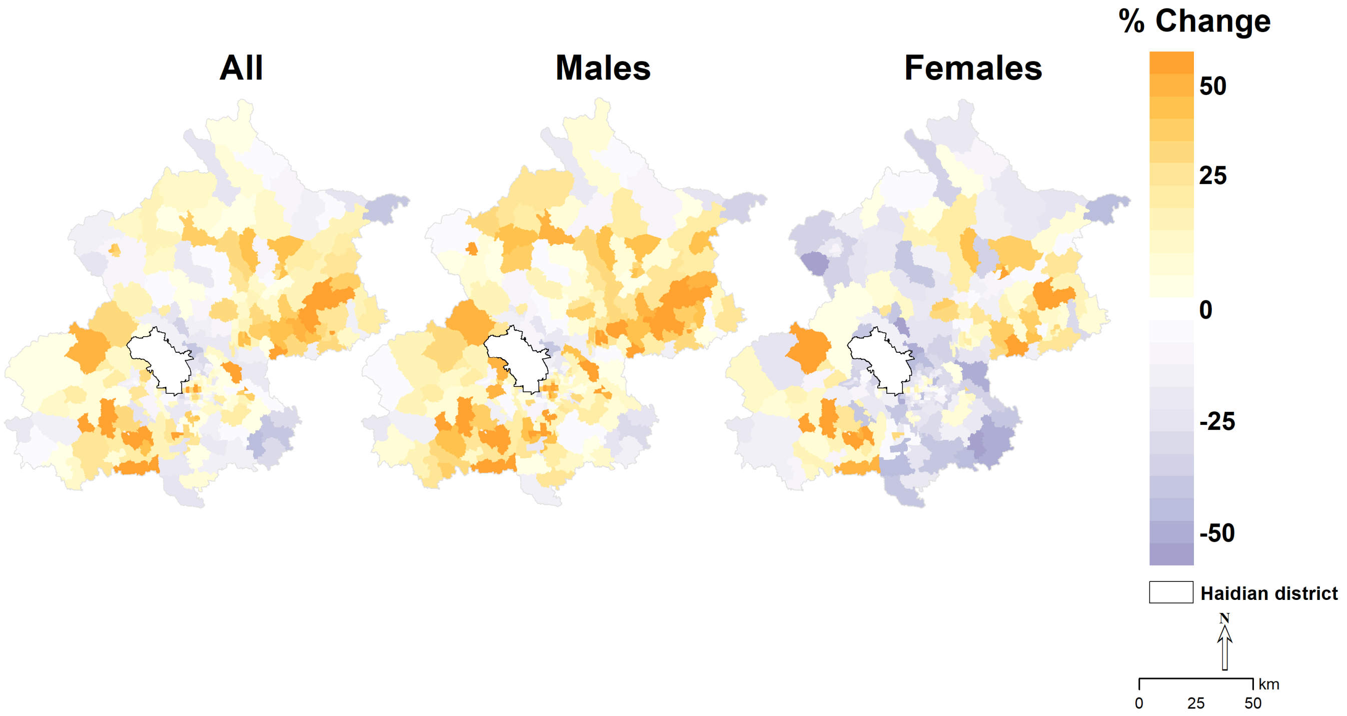

Figure S7. Percentage Changes in the Age-Standardized Incidence of Acute Myocardial Infarction in Beijing Residents Aged ≥ 35 Years at the Township Level after Excluding Data from Haidian District, 2007–2018

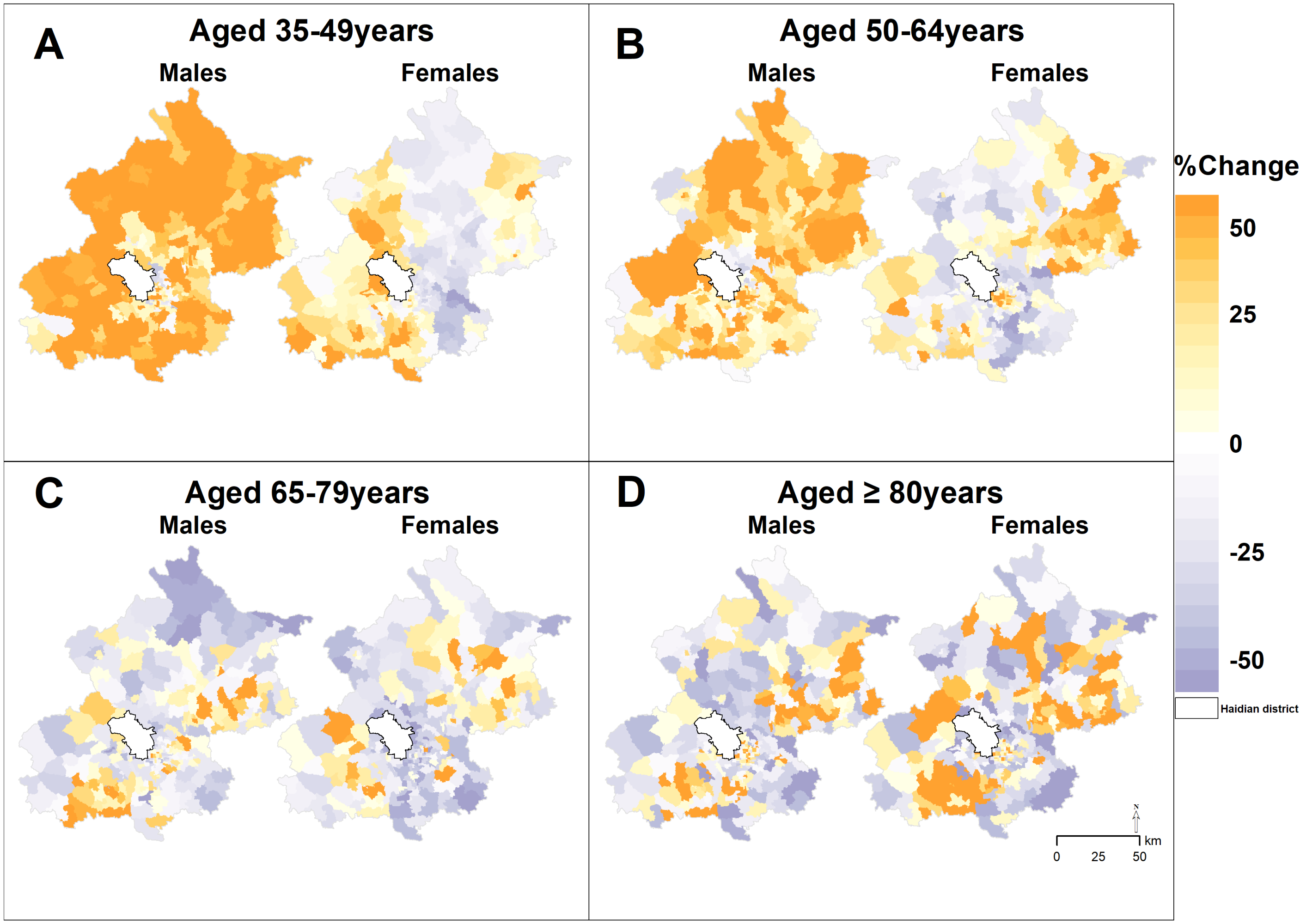

Figure S8. Percentage Changes in the Incidence of Acute Myocardial Infarction by Age-Sex Groups at the Township Level in Beijing after Excluding Data from Haidian District, 2007–2018

References

41. Zhang, Q.; Zhao, D.; Xie, W.; Xie, X.; Guo, M.; Wang, M.; Wang, W.; Liu, W.; Liu, J. Recent trends in hospitalization for acute myocardial infarction in Beijing: Increasing overall burden and a transition from ST-segment elevation to non-ST-segment elevation myocardial infarction in a population-based study. *Medicine (Baltimore)* **2016**, 95, e2677.

42. Besag, J.; York, J.; Mollié, A. Bayesian image restoration, with two applications in spatial statistics. *Ann. Inst. Stat. Math.* **1991**, 43, 1–20.
